# Effects of FABP5 Expression on Clinicopathological and Survival Characteristics in Digestive System Malignancies: A Systematic Review and Meta‐Analysis

**DOI:** 10.1002/cam4.70794

**Published:** 2025-04-03

**Authors:** Miaoqing Li, Xiaoxia Wang, Jia Guo, Junchen Qu, Yu Cao, Qingkun Song, Jun Lu

**Affiliations:** ^1^ Department of Medical Oncology, Laboratory for Clinical Medicine Beijing YouAn Hospital, Capital Medical University Beijing China; ^2^ Department of Clinical Epidemiology Research Beijing YouAn Hospital, Capital Medical University Beijing China

**Keywords:** clinicopathological features, digestive system malignancies, FABP5, fatty acid binding protein 5, meta‐analysis, survival analysis

## Abstract

**Background:**

Digestive system malignancies are a major global health burden, and the role of fatty acid binding protein 5 (FABP5) in these tumors remains controversial.

**Aims:**

This meta‐analysis aimed to evaluate the correlation between FABP5 expression and clinicopathological features, as well as survival outcomes in digestive system malignancies.

**Materials and Methods:**

Data from 11 studies (1207 patients) retrieved from PubMed, Embase, Cochrane Library, CNKI, and WanFang were analyzed.

**Results:**

FABP5 overexpression was associated with poorer overall survival (OS), larger tumor size, advanced UICC stage, and increased risk of vascular invasion and lymph node metastasis. Notably, FABP5 overexpression is particularly associated with poorer OS in the subgroup of digestive tract malignancies and larger tumor sizes in the subgroup of Chinese patients.

**Discussion:**

Cellular experiments demonstrated that FABP5 overexpression enhances proliferation, migration, and invasion in hepatocellular carcinoma (Huh7) and gastric cancer (HGC‐27) cell lines, while FABP5 knockdown reduces these effects. Mechanistically, FABP5 may drive tumor progression through PPARβ/δ signaling, epithelial‐mesenchymal transition induction, angiogenesis regulation, and potential effects on fatty acid metabolism and hypoxia‐related pathways.

**Conclusion:**

FABP5 overexpression correlates with adverse clinicopathological features and prognosis in digestive system malignancies, suggesting its potential as a biomarker for these tumors. Further research is warranted.

## 1 Introduction

Digestive system cancers exhibit high incidence and mortality rates worldwide, making them a major global health burden. This category includes malignancies of the esophagus, stomach, liver, bile ducts, gallbladder, pancreas, small intestine, colorectum, and anorectal region [1]. Studies have shown that different types of cancers share many common features in terms of chronic inflammation, genetic mutations, and epigenetic changes, imbalances in cell proliferation and apoptosis, as well as clinical manifestations [2]. For instance, both hepatocellular carcinoma (HCC) and gastric cancer (GC) often exhibit abnormal activation of the Wnt/β‐catenin signaling pathway and mutations in the TP53 gene, which promote tumor progression [3, 4]. Clinically, these cancers typically present with symptoms related to the digestive system. According to the latest data from the Global Cancer Database (Globocan) in 2022, approximately 4 million new cases of digestive system cancers arise annually, accounting for about 20% of global cancer incidence. The annual death toll exceeds 3 million cases, representing more than 30% of global cancer mortality. Colorectal cancer ranks as the third most common cancer worldwide and is the second leading cause of cancer‐related deaths. Liver cancer is the sixth most prevalent cancer globally, with the third highest mortality rate among cancer‐related deaths [5]. Therefore, exploring the pathophysiological mechanisms underlying digestive system cancers and identifying molecular biomarkers for early diagnosis are crucial steps in predicting patient prognosis, guiding personalized treatment, and developing novel therapeutic strategies.

Fatty acid binding protein 5 (FABP5), also known as Epidermal‐type Fatty Acid Binding Protein (E‐FABP), is a member of the fatty acid binding proteins (FABPs) family. FABPs are intracellular lipid‐binding proteins that play a crucial role in binding and transporting long‐chain fatty acids within cells [6]. These proteins serve as key regulatory factors in fatty acid metabolism, inflammation, and energy balance [7]. Research indicates that FABP5 is a target gene involved in the regulation of tumorigenesis and tumor progression in tumor cells influenced by both saturated and unsaturated fatty acids [8]. FABP5 can increase the expression of proteins involved in tumorigenesis by activating transcription factors (TFs), thereby promoting the growth and spread of tumors in various locations, such as lung [9], kidney [10], brain [11], and breast [12], especially closely related to various malignant tumors of the digestive system organs. In esophageal cancer [13, 14], gastric cancer [15, 16], liver cancer [17, 18, 19, 20, 21, 22], and colorectal cancer [23], studies have observed FABP5 overexpression, which correlates with tumor proliferation, invasiveness, and poor patient prognosis. However, some studies report contrasting findings, such as reduced FABP5 expression levels in pancreatic cancer [24], and evidence suggesting that FABP5 overexpression in liver cancer is associated with prolonged OS and disease‐free survival (DFS) [25].

Currently, the pathological mechanisms by which FABP5 contributes to the development of digestive system malignancies remain poorly understood, and existing studies yield inconsistent findings. To address this gap, we conducted a meta‐analysis of available studies to examine the impact of FABP5 expression on the clinicopathological features and prognosis of digestive system tumors.

## 2 Methods

### 2.1 Search Strategy

We conducted this study in accordance with the Preferred Reporting Items for Systematic Reviews and Meta‐Analyses (PRISMA) guidelines and registered it with PROSPERO (ID: CRD42024567624). We searched five major electronic databases—PubMed, Embase, Cochrane Library, CNKI, and WanFang—for relevant English‐language studies published up to August 2024. After the electronic search, we performed a manual search by cross‐referencing key papers to identify additional relevant studies. All retrieved materials were managed using EndNote 21. The search terms included “Digestive system malignancies,” “Digestive system cancers,” and “Digestive system neoplasms,” combined with “Fatty acid binding protein 5” and “FABP5.” The detailed search strategy is shown in Appendix 1.

### 2.2 Criteria for Inclusion and Exclusion

We included studies if they met the following eligibility criteria: [1] The full text of the study was available [2]. Patients were clearly diagnosed with digestive system cancer, and FABP5 expression was directly examined using immunohistochemistry (IHC) or RT‐PCR [3]. The study provided clinicopathological characteristics or survival data [4]. Hazard ratios (HRs) for survival data were reported or could be calculated from the published data.

We excluded studies if they met any of the following criteria: [1] Ineligible study types, including ecological studies, case reports, reviews, editorials, letters, conference abstracts, or animal trials; [2] Repeated studies based on the same database or patient cohort; [3] Studies that lacked sufficient data.

### 2.3 Data Extraction and Quality Assessment

Two investigators, Miaoqing Li and Xiaoxia Wang, independently screened the studies to determine whether the relevant articles met the inclusion criteria. We resolved any discrepancies through discussion, re‐extraction of data, or third‐party adjudication. The extracted data included the first author's name, publication year, number of patients, region of origin, sample source, assessment method, and clinicopathological features such as gender, age, UICC stage, tumor size, tumor number, tumor differentiation, vascular invasion, nerve invasion, lymph node metastasis, as well as survival data including HRs with 95% confidence intervals (CIs) for OS and DFS. We utilized the Newcastle‐Ottawa Scale (NOS) and Grading of Recommendations Assessment, Development and Evaluation (GRADE) to evaluate the quality of the included studies. Studies scoring NOS ≥ 6 were considered high quality, while those scoring below were regarded as poor quality. We included only high‐quality studies in this meta‐analysis.

### 2.4 Statistical Analysis

We used STATA 15.0 software to calculate pooled ORs and HRs with 95% CIs. We extracted survival data from Kaplan–Meier curves using Engauge Digitizer 12.1 software. We assessed the association between positive FABP5 expression and clinicopathological features using fixed or random effects models, depending on whether *I* ^2^ was less than or greater than 50%. An HR or OR greater than 1 indicated a worse prognosis for the group with FABP5 overexpression. We considered the results statistically significant if the 95% CI did not include 1.

## 3 Results

### 3.1 Eligible Studies

**FIGURE 1:**
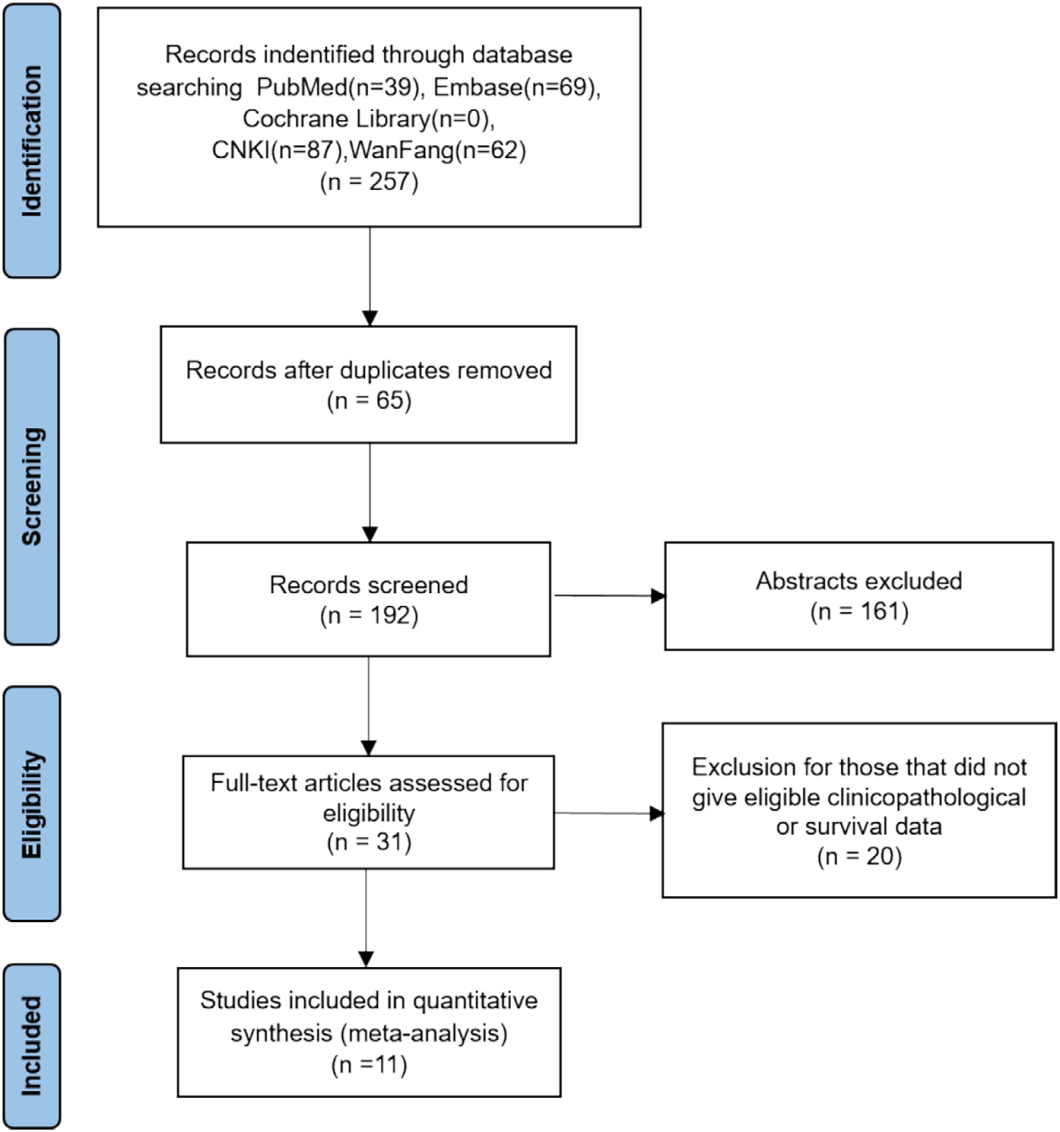
PRISMA flow chart of the search and selection procedure.

The retrieval process followed the PRISMA flow chart (Figure 1). Initially, we identified 257 studies based on the selection criteria described in the Methods section, including all digestive system cancers of the esophagus, stomach, colorectal, pancreas, liver, and bile duct. Of these, 65 studies were excluded due to duplication, and 161 were excluded after screening the summaries. Following a full‐text review, 20 additional articles were excluded due to missing data. Ultimately, 11 studies met the inclusion criteria and were selected for analysis, which were related to esophageal cancer, gastric cancer, cholangiocarcinoma, and liver cancer.

### 3.2 Main Characteristics and Quality Assessment of the Included Studies

Eleven studies with 1207 patients were included for meta‐analysis (Table 1). These studies originated from three countries (China, Korea, and Japan) and were published from 2018 to 2023. Sample sizes ranged from 38 to 344, and the NOS scale scores of all the studies were ≥ 6, suggesting high quality; the specific scoring details are presented in Appendix 2. The GRADE system was utilized to assess the credibility of the included studies. Owing to the inherent limitations of the included studies being nonrandomized controlled trials, the results generally demonstrated a low level of credibility. The detailed scoring situation is displayed in Appendix 3.

**TABLE 1:** Main characteristics of studies included in the meta‐analysis.

Authors	Year	Country	Tumor	No. of patients	Sample	Assessment method	Survival analysis	HRs of survival analysis	Gender (Male vs. Female)	Age (> 60 vs. < 60)	UICC stage (III + IV vs. I + II)	Tumor size (> 5 cm vs. ≤ 5 cm)	Tumor number (multinodular vs. single)	Tumor differentiation (moderate and high vs. low)	Vascular invasion (present vs. absent)	Nerve invasion (present vs. absent)	Lymph node metastasis (present vs. absent)	NOS score
Chen et al.	2022	China	ESCC	56	tissue	IHC	OS	Estimate	43/13	NR	NR	NR	NR	NR	NR	NR	NR	8
Li et al.	2021	China	ESCC	344	tissue	IHC	NR	NR	119/53	83/58	99/102	116/56	NR	137/35	33/139	35/137	57/115	8
Qiu et al.	2023	China	GC	104	tissue	IHC	OS	Reported in article	55/24	NR	NR	NR	NR	59/20	NR	NR	57/22	8
Wang et al.	2021	China	GC	101	tissue	IHC	OS	Reported in article	70/31	NR	62/38	NR	NR	53/48	48/20	37/35	77/24	7
Jeong et al.	2012	Korea	ICC	59	tissue	IHC	NR	NR	NR	NR	10/33	28/21	22/35	34/9	6/37	8/35	8/35	6
Liu et al.	2022	China	HCC	76	tissue	IHC	OS	Reported in article	57/19	40/32	35/41	36/40	NR	NR	53/53	NR	NR	7
Liu et al.	2020	China	HCC	118	tissue	IHC	OS/DFS	Estimate	NR	NR	NR	NR	23/53	42/34	NR	NR	NR	7
Ohira et al.	2021	Japan	HCC	220	tissue	WB	OS/DFS	Estimate	107/21	NR	59/69	59/71	72/92	101/27	50/78	NR	NR	7
Pan et al.	2018	China	HCC	43	tissue	IHC	NR	NR	35/8	NR	27/28	21/22	NR	31/12	22/21	NR	NR	6
Tang et al.	2022	China	HCC	48	tissue	IHC	NR	NR	21/13	16/32	NR	36/12	NR	19/29	NR	NR	15/33	7
Seo et al.	2020	Korea	HCC	38	tissue	IHC	DFS	Estimate	NR	NR	NR	NR	NR	NR	NR	NR	NR	8

Abbreviations: DFS, disease‐free survival; ESCC, esophageal squamous cell carcinoma; GC, gastric carcinoma; HCC, Hepatocellular carcinoma; ICC, intrahepatic cholangiocarcinoma; IHC, immunohistochemistry; NOS, Newcastle‐Ottawa Quality Assessment Scale; NR, not reported; OS, overall survival; WB, Western Blotting.

### 3.3 Association Between FABP5 Expression and all Characteristics in Patients With Digestive System Cancer

**FIGURE 2:**
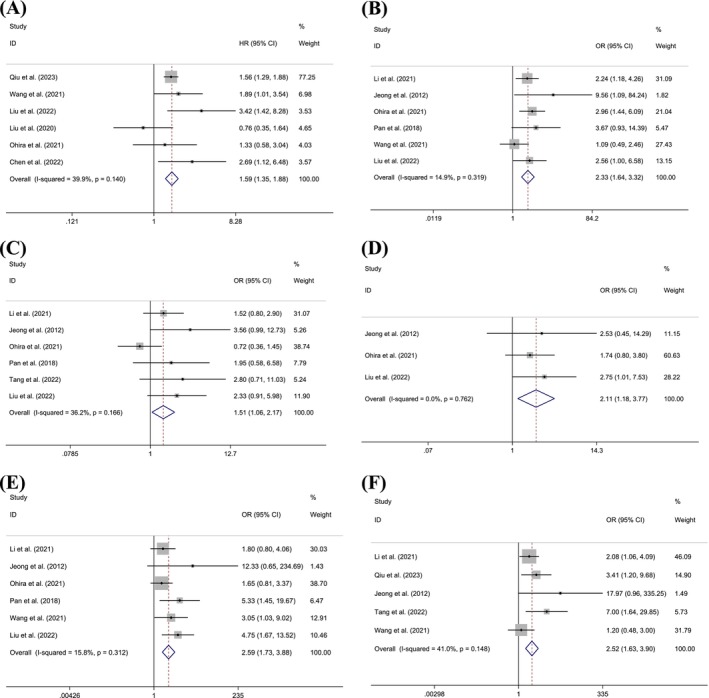
Forest plot of survival and clinicopathological characteristics with FABP5 expression, including (A) OS, (B) UICC stage, (C) tumor size, (D) tumor number, (E) vascular invasion, and (F) lymph node metastasis.

We combined the HRs for survival characteristics (Table 2). Six studies involving 558 patients demonstrated a significant correlation between FABP5 expression and OS (OR = 1.592, 95% CI = 1.349, 1.880, *p* = 0.000, *I* ^2^ = 39.9%) (Figure 2A). In contrast, three studies with 284 HCC patients showed no significant correlation between FABP5 expression and DFS (*p* > 0.05).

**TABLE 2:** Survival and clinicopathological characteristics and pooled HRs/ORs associated with FABP5 expression.

Characteristics	No. of studies	No. of patients	Pooled HR/OR (95% CI)	*p*	Heterogeneity	
					*I* ^2^ (%)	*p*
Overall survival	6	558	1.592 (1.349, 1.880)	< 0.001	39.9	0.140
Disease‐free survival	3	284	1.049 (0.529, 2.081)	0.891	69.6	0.037
Gender (Male vs. Female)	8	689	0.782 (0.554, 1.104)	0.163	0.0	0.769
Age (> 60 years vs. < 60 years)	3	261	0.923 (0.569, 1.498)	0.747	21.0	0.282
UICC stage (III + IV vs. I + II)	6	603	2.331 (1.637, 3.319)	< 0.001	14.9	0.319
Tumor size (> 5 cm vs. ≤ 5 cm)	6	518	1.515 (1.056, 2.174)	0.024	36.2	0.166
Tumor number (multinodular vs. single)	3	297	2.114 (1.185, 3.774)	0.011	0.0	0.762
Tumor differentiation (moderate and high vs. low)	8	690	0.722 (0.361, 1.443)	0.356	70.7	0.001
Vascular invasion (present vs. absent)	6	560	2.590 (1.729, 3.881)	< 0.001	15.8	0.312
Nerve invasion (present vs. absent)	3	287	0.985 (0.571, 1.700)	0.958	0.0	0.485
Lymph node metastasis (present vs. absent)	5	443	2.518 (1.626, 3.900)	< 0.001	41.0	0.148

We also combined the ORs for clinicopathological characteristics (Table 2). FABP5 expression significantly correlated with UICC stage (OR = 2.331, 95% CI = 1.637, 3.319, *p* < 0.001, *I* ^2^ = 14.9%) (Figure 2B), tumor size (OR = 1.515, 95% CI = 1.056, 2.174, *p* = 0.024, *I* ^2^ = 36.2%) (Figure 2C), tumor number (OR = 2.114, 95% CI = 1.185, 3.774, *p* = 0.011, *I* ^2^ = 0.0%) (Figure 2D), vascular invasion (OR = 2.590, 95% CI = 1.729, 3.881, *p* < 0.001, *I* ^2^ = 15.8%) (Figure 2E), and lymph node metastasis (OR = 2.518, 95% CI = 1.626, 3.900, *p* < 0.001, *I* ^*2*^ = 41.0%) (Figure 2F). However, FABP5 expression was not significantly associated with gender, age, tumor differentiation, or nerve invasion (*p* > 0.05).

### 3.4 Subgroup Analysis of Heterogeneous Characteristics

We performed subgroup analyses for variables with heterogeneity greater than 50%, such as disease‐free survival and tumor differentiation. Although heterogeneity in other pathological characteristics was minimal, we conducted further subgroup analyses due to differences in specific tumor names and types and countries of origin across the included studies (Table 3).

**TABLE 3:** Subgroup analysis of characteristics and pooled HRs/ORs associated with FABP5 expression.

Subgroup	No. of studies	No. of patients	Pooled HR/OR (95% CI)	*p*	Heterogeneity	
					*I* ^2^ (%)	*p*
Overall survival	6	558	1.592 (1.349, 1.880)	< 0.001	39.9	0.140
Tumor name						
GC	2	180	1.585 (1.323, 1.899)	< 0.001	0.0	0.570
ESCC	1	56	2.690 (1.117, 6.475)	—	—	—
HCC	3	322	1.480 (0.632, 3.466)	0.366	68.6	0.041
Tumor type						
Digestive tract	3	236	1.619 (1.356, 1.933)	< 0.001	0.0	0.436
Digestive gland	3	322	1.480 (0.632, 3.466)	0.366	68.6	0.041
Country						
China	5	430	1.723 (1.174, 2.526)	0.005	50.8	0.087
Others	1	128	1.330 (0.582, 3.040)	—	—	—
Disease‐free survival	3	284	1.049 (0.529, 2.081)	0.891	69.6	0.037
Country						
China	1	118	1.200 (0.791, 1.821)	—	—	—
Others	2	166	1.056 (0.261, 4.264)	0.939	79.4	0.027
UICC stage	6	603	2.331 (1.637, 3.319)	< 0.001	14.9	0.319
Tumor name						
GC	1	101	1.093 (0.486, 2.456)	—	—	—
ESCC	1	344	2.244 (1.181, 4.263)	—	—	—
ICC	1	43	9.563 (1.085, 84.243)	—	—	—
HCC	3	259	2.920 (1.721, 4.953)	< 0.001	0.0	0.913
Tumor type						
Digestive tract	3	344	1.984 (0.876, 4.496)	0.101	51.9	0.125
Digestive gland	3	259	2.920 (1.721, 4.953)	< 0.001	0.0	0.913
Country						
China	4	432	1.989 (1.310, 3.020)	0.001	8.5	0.351
Others	2	171	3.486 (1.777, 6.838)	< 0.001	2.4	0.312
Tumor size	6	790	1.515 (1.056, 2.174)	0.024	36.2	0.166
Tumor name						
ICC	1	43	3.556 (0.993, 12.733)	—	—	—
ESCC	1	344	1.524 (0.800, 2.902)	—	—	—
HCC	4	387	1.545 (0.760, 3.141)	0.229	48.8	0.119
Tumor type						
Digestive tract	2	403	1.932 (0.916, 4.075)	0.084	26.0	0.245
Digestive gland	4	387	1.545 (0.760, 3.141)	0.229	48.8	0.119
Country						
China	4	511	1.870 (1.182, 2.959)	0.008	0.0	0.816
Others	2	279	1.457 (0.307, 6.913)	0.636	36.2	0.166
Tumor number	3	297	2.114 (1.185, 3.774)	0.011	0.0	0.762
Tumor name						
ICC	1	57	2.526 (0.447, 14.293)	—	—	—
HCC	2	240	2.063 (1.115, 3.817)	0.021	0.0	0.481
Country						
China	1	76	2.753 (1.007, 7.529)	—	—	—
Others	2	221	1.863 (0.917, 3.785)	0.085	0.0	0.701
Tumor differentiation	8	995	0.722 (0.361, 1.443)	0.356	70.7	0.001
Tumor name						
GC	2	205	0.577 (0.275, 1.211)	0.146	25.7	0.246
ESCC	1	344	1.941 (0.918, 4.106)	—	—	—
ICC	1	43	0.125 (0.014, 1.111)	—	—	—
HCC	4	387	0.719 (0.220, 2.352)	0.585	77.4	0.004
Tumor type						
Digestive tract	4	608	0.705 (0.268, 1.855)	0.479	71.7	0.014
Digestive gland	4	387	0.719 (0.220, 2.352)	0.585	77.4	0.004
Country						
China	6	716	0.941 (0.443, 1.997)	0.874	70.4	0.005
Others	2	279	0.329 (0.144, 0.750)	0.008	0.0	0.349
Vascular invasion	6	560	2.590 (1.729, 3.881)	< 0.001	15.8	0.312
Tumor name						
ICC	1	43	12.333 (0.648, 234.685)	—	—	—
GC	1	101	3.051 (1.032, 9.022)	—	—	—
ESCC	1	344	1.799 (0.797, 4.062)	—	—	—
HCC	3	277	2.659 (1.566, 4.514)	< 0.001	49.9	0.136
Tumor type						
Digestive tract	3	283	2.504 (1.340, 4.677)	0.004	0.0	0.389
Digestive gland	3	277	2.659 (1.566, 4.514)	< 0.001	49.9	0.136
Country						
China	4	389	2.966 (1.785, 4.930)	< 0.001	0.2	0.391
Others	2	171	2.029 (1.035, 3.978)	0.039	43.4	0.184
Nerve invasion	3	287	0.985 (0.571, 1.700)	0.958	0.0	0.485
Country						
China	2	244	0.796 (0.377, 1.682)	—	—	—
Others	1	43	1.254 (0.562, 2.795)	0.580	0.0	0.356
Lymph node metastasis	5	656	2.518 (1.626, 3.900)	< 0.001	41.0	0.148
Tumor name						
GC	2	205	1.961 (0.706, 5.446)	0.196	53.9	0.2938
ESCC	1	344	2.083 (1.060, 4.092)	—	—	—
ICC	1	43	17.971 (0.963, 335.249)	—	—	—
HCC	1	48	7.000 (1.641, 29.854)	—	—	—
Tumor type						
Digestive tract	4	608	2.194 (1.179, 4.083)	0.013	30.6	0.228
Digestive gland	1	48	7.000 (1.641, 29.854)	—	—	—
Country						
China	4	597	2.383 (1.294, 4.388)	0.005	37.7	0.186
Others	1	59	17.971 (0.963, 335.249)	—	—	—

**FIGURE 3:**
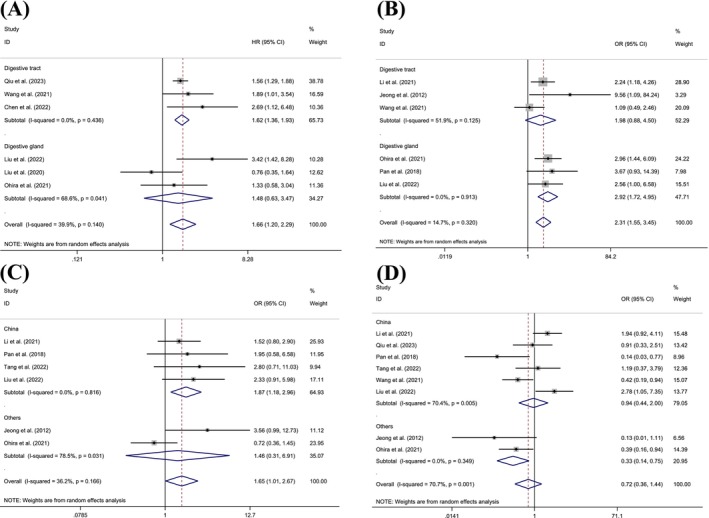
Subgroup analysis of forest plots of characteristics with FABP5 expression, including (A) OS of different tumor types, (B) UICC stages of different tumor types, (C) tumor sizes of different countries, and (D) tumor differentiation of different countries.

Six studies involving 558 patients showed that FABP5 expression was more strongly associated with OS in patients with digestive tract cancers (OR = 1.619, 95% CI = 1.356, 1.933, *p* < 0.001, *I* ^2^ = 0.0%) especially in GC (OR = 1.585, 95% CI = 1.323, 1.899, *p* < 0.001, *I* ^2^ = 0.0%), compared to patients with HCC (Table 3, Figure 3A). Additionally, given that all digestive tract patients (GC and ESCC) with available OS clinical data were derived from China, the results may hold greater validity within the Chinese patient population. Six studies with 603 patients revealed that FABP5 expression was more strongly associated with UICC in patients with HCC (OR = 2.920, 95% CI = 1.721, 4.953, *p* < 0.001, *I* ^2^ = 0.0%) compared to those with digestive tract cancers, including GC, ESCC, and ICC (Table 3, Figure 3B).

Furthermore, six studies with 790 patients demonstrated that FABP5 expression was more significantly linked to tumor size in patients from China (OR = 1.870, 95% CI = 1.182, 2.959, *p* = 0.008, *I* ^2^ = 0.0%) compared to those from other countries (Table 3, Figure 3C). Although eight studies involving 995 patients indicated that FABP5 expression was significantly associated with tumor differentiation in patients from countries other than China (specifically Korea and Japan) (OR = 0.329, 95% CI = 0.144, 0.750, *p* = 0.008, *I* ^2^ = 0.0%), the significance of this finding is limited by the geographic variations (Table 3, Figure 3D).

### 3.5 Publication Biases and Sensitivity Analysis

**FIGURE 4:**
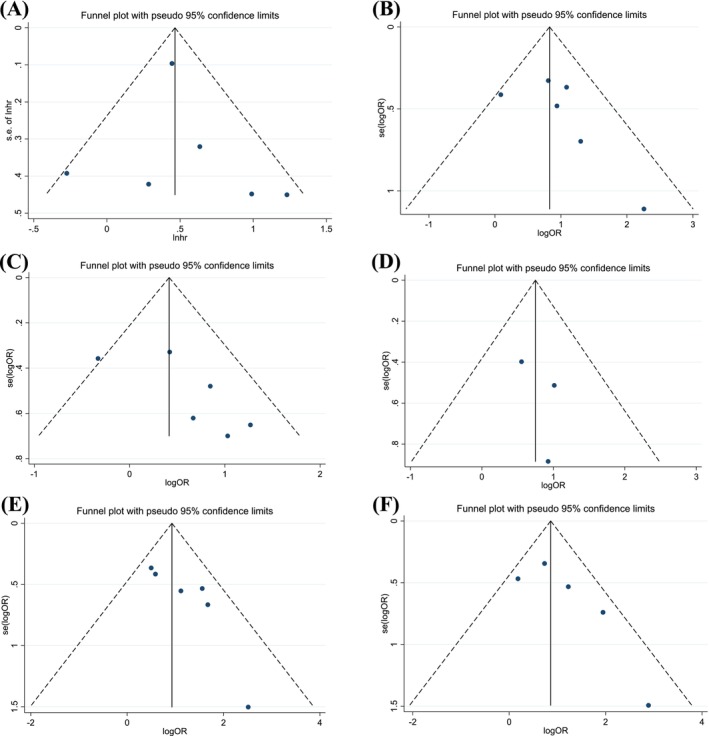
Funnel plots of survival and clinicopathological characteristics, including (A) OS, (B) UICC stage, (C) tumor size, (D) tumor number, (E) vascular invasion, and (F) lymph node metastasis.

We employed Funnel plots, Egger's tests, and Begg's tests to assess potential publication bias in the data for each characteristic in patients with digestive system cancer. For characteristics with significant differences, the Funnel plots showed a largely symmetrical distribution (Figure 4), suggesting minimal bias. Further quantitative analysis using Egger's and Begg's tests revealed no significant publication bias (*p* > 0.05) for most characteristics (Table 4), with one exception. In the case of vascular invasion, Egger's test suggested possible bias; however, this was not supported by the results of the Begg's test or the Funnel plots.

**TABLE 4:** Begg's and Egger's tests performed for characteristics with significant associations.

Characteristics	*P*_B_	*P*_E_
OS	0.133	0.702
UICC stage	0.260	0.278
Tumor size	0.260	0.109
Tumor number	1.000	0.584
Vascular invasion	0.260	0.039
Lymph node metastasis	0.221	0.142

*Note:* *P*_B_ *P* value of the Begg's rank correlation test, *P*_E_ *P* value of the Egger's linear regression test.

**FIGURE 5:**
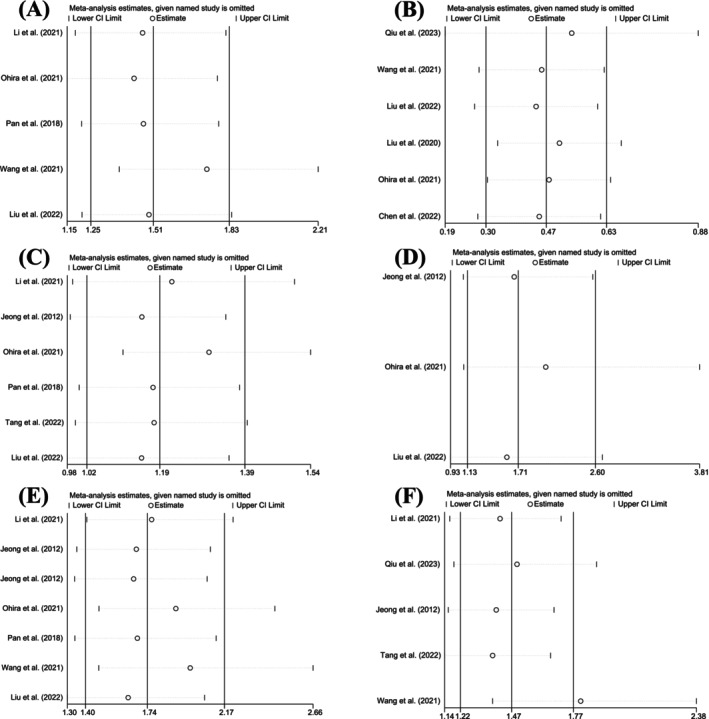
Sensitivity analysis of survival and clinicopathological characteristics, including (A) OS, (B) UICC stage, (C) Tumor size, (D) Tumor number, (E) Vascular invasion, and (F) lymph node metastasis.

We conducted a sensitivity analysis by excluding each article one by one (Figure 5). The results indicated that the overall findings were not significantly affected by the removal of any individual study, suggesting that our results are reliable.

## 4 Discussion

This systematic review and meta‐analysis was conducted using rigorous inclusion and exclusion criteria, compiled clinical–pathological data and survival information from 1207 patients across 11 retrospective cohort studies. To our knowledge, this is the first systematic review and meta‐analysis to evaluate the impact of FABP5 expression on clinicopathological features and survival outcomes in digestive system malignancies. Regarding the strength of evidence, the NOS assessment of the included studies demonstrated moderate to high quality. However, due to the nature of retrospective evidence, the GRADE system rated the evidence quality as moderate or lower. Therefore, there is a need to incorporate more prospective studies.

Our findings indicate that patients with digestive system tumors who exhibit overexpression of FABP5 have poorer survival outcomes. Specifically, FABP5 overexpression significantly correlates with OS among these patients, and subsequent subgroup analyses reveal a stronger association in patients with digestive tract tumors, especially in GC. We observed substantial heterogeneity in DFS among patients with HCC (*I* ^2^ = 69.6%), with no significant correlation to FABP5 expression. Further subgroup analyses did not reduce this heterogeneity, which remained high (*I* ^2^ = 79.4%). This high heterogeneity may stem from the limited number of studies included and the variability in HCC staging and treatment approaches among the patients. For instance, Liu et al. [25] included studies that enrolled patients who had not received radiotherapy or chemotherapy prior to tumor resection but did not restrict those with comorbidities; Ohira et al. [19] excluded patients with preoperative alcoholic hepatitis or autoimmune diseases; and Seo et al. [22] did not limit tumor staging and made no mention of restrictions on treatment or comorbidities. Similar scenarios were observed in the analysis of the correlation between FABP5 expression and all other indicators, where the inclusion and exclusion criteria for patients varied across studies or were not specified. Although the pooled analysis exhibited low heterogeneity and both sensitivity and bias analyses indicated robust results with a certain level of credibility, the potential impact of confounding factors should still be taken into account. Additionally, the patients with available DFS data were exclusively those with HCC, which limits the generalizability of the findings to all patients with digestive system cancers. Similar observations apply to the conclusions regarding OS. Our study demonstrated a stronger correlation between OS and FABP5 expression in patients with digestive tract tumors, particularly in those with GC. However, all patients with digestive tract tumors included in the analysis were sourced from China. These limited factors collectively suggest that future analyses incorporating a larger number of studies are necessary.

Our study reveals that patients with digestive system malignancies and elevated FABP5 expression exhibit more severe clinical–pathological features. These include advanced UICC stage, larger tumor size, greater tumor numbers, and an increased propensity for vascular invasion and lymph node metastasis. Subgroup analyses further indicate that FABP5 expression is particularly associated with tumor size in the Chinese patient subgroup, as well as with UICC stage in the subgroup of patients with HCC. While FABP5 expression demonstrated a stronger correlation with tumor differentiation in two studies conducted outside of China (specifically in Korea and Japan), the significance of this finding is diminished by the differing geographical origins of these studies. This suggests that the correlation between FABP5 expression and tumor differentiation may not be evident in studies conducted in China. Therefore, further research that includes a broader range of studies is warranted to enhance our understanding of this relationship.

Moreover, we conducted relevant cellular experiments to validate our conclusions; the relevant experimental methods are shown in Data S1. In the hepatocellular carcinoma cell line Huh7 and the gastric cancer cell line HGC‐27, overexpression of FABP5 enhanced cancer cell proliferation (Data S2A), migration (Data S2B), and invasion (Data S2C), whereas knockdown of FABP5 attenuated these capabilities (Data S3A–C). Multiple studies have identified various mechanisms through which FABP5 promotes tumor development, supporting our findings. FABP5 facilitates tumor cell proliferation and metastasis by modulating the PPAR β/δ signaling pathway and stabilizing epidermal growth factor [26]. It also promotes epithelial‐mesenchymal transition [27], with the PPAR β/δ signaling pathway serving as a primary mechanism [28, 29, 30]. Furthermore, FABP5 regulates the expression of VEGF through PPARγ [31] and enhances the expression of other angiogenic factors, including EGF [26] and IL‐6 [32], thereby promoting tumor angiogenesis. Additionally, FABP5 modulates the function of vascular endothelial cells; its deficiency impairs cell proliferation and chemotactic migration [33]. However, some studies indicate that the pro‐oncogenic mechanisms of FABP5 may function independently of the PPAR β/δ or PPAR γ signaling pathways, instead relying on the modulation of fatty acid metabolism within cancer cells [34]. FABP5 can activate hypoxia‐inducible factor‐1α (HIF‐1α) and is involved in regulating genes associated with lipid storage, such as acyl‐coenzyme A synthetase long chain family member 1 (ACSL1), glycerol‐3‐phosphate acyltransferase (GPAT), Lipin‐1, and diacylglycerol O‐acyltransferase 2 (DGAT2) [22]. Additionally, FABP5 may regulate genes involved in de novo fatty acid synthesis and fatty acid breakdown, including fatty acid synthase (FASN), stearoyl‐CoA desaturase‐1 (SCD1), and ATP citrate lyase (ACLY) [9].

Furthermore, FABP5 may contribute to inflammatory responses in tumor cells by modulating the expression of interleukin‐1 (IL‐1), IL‐6, and IL‐8, as well as parathyroid hormone‐related protein. This modulation leads to the production of reactive oxygen species (ROS) and the activation of protein kinase C [35]. FABP5 may also promote tumor cell energy production by regulating the AMP to ADP/ATP ratio, protein kinases, and the number of autophagosomes [36].

## 5 Conclusion

Our study confirms that the overexpression of FABP5 is associated with poorer clinical–pathological features and reduced overall survival in patients with digestive system tumors, as demonstrated through meta‐analysis. This finding highlights the significant role of FABP5 in exploring the pathophysiological mechanisms of digestive system malignancies. However, due to the limited number of current studies and small sample sizes, further research with larger cohorts is necessary to provide additional support. FABP5 represents a valuable molecular biomarker for the treatment of digestive system cancers and requires further investigation in the future.

## Author Contributions

**Miaoqing Li:** conceptualization, methodology, software, data curation, formal analysis, writing – original draft, writing – review and editing, validation, investigation, visualization, supervision, resources, project administration. **Xiaoxia Wang:** methodology, software, validation, investigation. **Jia Guo:** validation, resources. **Junchen Qu:** data curation. **Yu Cao:** writing – review and editing, validation. **Qingkun Song:** validation. **Jun Lu:** funding acquisition, writing – review and editing, resources.

## Ethics Statement

The authors have nothing to report.

## Consent

The authors have nothing to report.

## Conflicts of Interest

The authors declare no conflicts of interest.

## Supporting information


Data S1.

Data S2.

Data S3.

## Data Availability

All data needed to support the conclusions are presented in this paper. Additional data related to this study were obtained from the authors.

## Appendix 1

### Pubmed

((((FABP5 protein, human[MeSH Terms]) OR (E‐FABP protein, human[Title/Abstract])) OR (fatty acid binding protein 5 (psoriasis‐associated), human[Title/Abstract])) OR (Fatty acid‐binding protein, epidermal, human[Title/Abstract])) AND ((((((((((Digestive System Neoplasms[MeSH Terms]) OR (Digestive System Neoplasm[Title/Abstract])) OR (Neoplasm, Digestive System[Title/Abstract])) OR (Neoplasms, Digestive System[Title/Abstract])) OR (Cancer of Digestive System[Title/Abstract])) OR (Digestive System Cancers[Title/Abstract])) OR (Cancer of the Digestive System[Title/Abstract])) OR (Digestive System Cancer[Title/Abstract])) OR (Cancer, Digestive System[Title/Abstract])) OR (Cancers, Digestive System[Title/Abstract])).

### Embase

(“digestive system cancer”/exp OR “digestive system cancer” OR “alimentary canal cancer”:ab,ti OR “alimentary cancer”:ab,ti OR “alimentary malignancies”:ab,ti OR “alimentary malignancy”:ab,ti OR “alimentary tract cancer”:ab,ti OR “cancer in the digestive tract”:ab,ti OR “cancer of the alimentary tract”: ab,ti OR “cancer of the digestive tract”:ab,ti OR “carcinogenesis in the digestive tract”:ab,ti OR “digestive canal cancer”:ab,ti OR “digestive cancer”:ab,ti OR “digestive malignancies”:ab,ti OR “digestive malignancy”:ab,ti OR “digestive system cancer”:ab,ti OR “digestive tract carcinogenesis”:ab,ti OR “digestive tract malignancies”:ab,ti OR “digestive tract malignancy”:ab,ti OR “malignancies of the digestive tract”:ab,ti OR “malignancy of digestive tract”:ab,ti OR “digestive system cancer”:ab,ti) AND “fabp5”:ab,ti.

### CNKI and WanFang

The key subject word “tumor” is combined with the phrase “FABP5” or “fatty acid binding protein 5” contained in the abstract. The retrieval strategy uses Chinese terms.

## Appendix 2 Detailed Score Information of Each Study According to the NOS Scale

**Table:**

Criteria		Li 2021	Qiu 2023	Wang 2021	Jeong 2021	Liu 2022	Liu 2020	Ohira 2021	Pan 2018	Tang 2022	Seo 2020	Chen 2022
Selection	Representativeness of the exposed cohort	1	1	1	1	1	1	1	1	1	1	1
	Selection of the non‐exposed cohort	1	1	1	1	1	1	1	1	1	1	1
	Ascertainment of exposure	1	1	1	1	1	1	1	1	1	1	1
	Demonstration that outcome of interest was not present at start of study	1	1	1	1	1	1	1	1	1	1	1
Comparability	Comparability of COHORTS on the basis of the design or analysis	2	1	1	1	1	2	2	1	2	2	2
Outcome	Assessment of outcome	1	1	1	1	1	0	0	1	1	0	0
	Was follow‐up long enough for outcomes to occur	1	1	0	0	0	0	0	0	0	1	1
	Adequacy of follow up of cohorts	0	1	1	0	1	1	1	0	0	1	1
Total scores		8	8	7	6	7	7	7	6	7	8	8

## Appendix 3 GRADE Summary of Studies Using Gradepro Software

**Table:**

Certainty assessment							№ of patients	Relative Effect (95% CI)	Certainty
№ of studies	Study design	Risk of bias	Inconsistency	Indirectness	Imprecision	Other considerations			
**Overall survival**									
6	Non‐randomised studies	Serious (Qiu 2023, Wang 2021, Liu 2022)^a^	Serious (Liu 2020)^b^	Not serious	Serious (Liu 2022)^c^	Strong association (Liu 2022, Chen 2022)^d^	558	**HR 1.592** (1.349 to 1.880)	⨁◯◯◯ Very low Qiu 2023, Wang 2021, Liu 2022, Liu 2020 ⨁⨁◯◯ Low Ohira 2021 ⨁⨁⨁◯ Moderate Chen 2022
**Disease‐free survival**									
3	Non‐randomised studies	Not serious	Serious (Liu 2020)^b^	Not serious	Serious (Seo 2020)^c^	None	284	**HR 1.049** (0.529 to 2.081)	⨁◯◯◯ Very low Liu 2020, Seo 2020 ⨁⨁◯◯ Low Ohira 2021
**Gender (Male vs. Female)**									
8	Non‐randomised studies	Serious (Qiu 2023, Wang 2021, Liu 2022, Pan 2018)^a^	Not serious	Not serious	Not serious	None	689	**OR 0.782** (0.554 to 1.104)	⨁◯◯◯ Very low Qiu 2023, Wang2021, Liu 2022, Pan 2018 ⨁⨁◯◯ Low Li 2021, Ohira 2021, Tang 2022, Chen 2022
**Age (> 60 years vs < 60 years)**									
3	non‐randomised studies	Serious (Liu 2022)^a^	Not serious	Not serious	Not serious	None	261	**OR 0.923** (0.569, 1.498)	⨁◯◯◯ Very low Liu 2022 ⨁⨁◯◯ Low Li 2021, Tang 2022
**UICC stage (III+IV vs I+II)**									
6	Non‐randomised studies	Serious (Jeong 2021, Wang 2021, Liu 2022, Pan 2018)^a^	Serious (Wang 2021)^b^	Not serious	Not serious	None	603	**OR 2.331** (1.637, 3.319)	⨁◯◯◯ Very low Jeong 2021, Wang 2021, Liu 2022, Pan 2018 ⨁⨁◯◯ Low Li 2021
**Tumor size (> 5cm vs ≤ 5cm)**									
6	non‐randomised studies	Serious (Jeong 2021, Liu 2022, Pan 2018)^a^	Serious (Jeong 2021)^b^	Not serious	Not serious	None	518	**OR 1.515** (1.056, 2.174)	⨁◯◯◯ Very low Jeong 2021, Liu 2022, Pan 2018 ⨁⨁◯◯ Low Li 2021, Tang 2022
**Tumor number (multinodular vs single)**									
3	Non‐randomised studies	Serious (Jeong 2021, Liu 2022)^a^	Serious (Liu 2022)^b^	Not serious	Not serious	None	297	**OR 2.114** (1.185, 3.774)	⨁◯◯◯ Very low Jeong 2021, Liu 2022 ⨁⨁◯◯ Low Ohira 2021
**Tumor differentiation (moderate and high vs low)**									
8	Non‐randomised studies	Serious (Qiu 2023, Jeong 2021, Wang 2021, Liu 2022, Pan 2018)^a^	Serious (Ohira 2021, Wang 2021, Liu 2022, Pan 2018)^b^	Not serious	Not serious	None	690	**OR 0.722** (0.361, 1.443)	⨁◯◯◯ Very low Ohira 2021, Qiu 2023, Jeong 2021, Wang 2021, Liu 2022, Pan 2018 ⨁⨁◯◯ Low Li 2021, Tang 2022
**Vascular invasion (present vs absent)**									
6	Non‐randomised studies	Serious (Jeong 2021, Wang 2021, Liu 2022, Pan 2018)^a^	Serious (Ohira 2021)^b^	Not serious	Not serious	None	560	**OR 2.590** (1.729, 3.881)	⨁◯◯◯ Very low Ohira 2021, Jeong 2021, Wang 2021, Liu 2022, Pan 2018 ⨁⨁◯◯ Low Li 2021
**Nerve invasion (present vs absent)**									
3	Non‐randomised studies	Serious (Jeong 2021, Wang 2021)^a^	Not serious	Not serious	Not serious	None	287	**OR 0.985** (0.571, 1.700)	⨁◯◯◯ Very low Jeong 2021, Wang 2021 ⨁⨁◯◯ Low Li 2021
**Lymph node metastasis (present vs absent)**									
3	Non‐randomised studies	Serious (Qiu 2023, Jeong 2021, Wang 2021)^a^	Serious (Wang 2021)^b^	Not serious	Not serious	None	287	**OR 0.985** (0.571, 1.700)	⨁◯◯◯ Very low Qiu 2023, Jeong 2021, Wang 2021 ⨁⨁◯◯ Low Li 2021, Tang 2022

Abbreviations: CI, confidence interval; HR, hazard ratio; OR, odds ratio.

^a^ No patient inclusion or exclusion criteria.

^b^ Results are inconsistent with others.

^c^ The confidence interval is excessively large.

^d^ The hazard ratio (HR) is greater than 2.0 or less than 0.5, and this result is supported by at least two consistent studies, with no credible confounding factors.
