# Supplementary material for: Effects of FABP5 Expression on Clinicopathological and Survival Characteristics in Digestive System Malignancies: A Systematic Review and Meta‐Analysis
Source: Cancer Med. 2025 Apr 3;14(7):e70794. doi: 10.1002/cam4.70794 (PMC11966564; doi:10.1002/cam4.70794)
Supplement: Supplementary file 1 — Data S1. [file CAM4-14-e70794-s003.docx]

**Cell culture and transfection.**

Huh7 and HGC-27 (Laboratory of Cardiovascular Electrophysiology, School of Basic Medical Sciences, Capital Medical University) were cultured with RPMI-1640, 10% fetal bovine serum (FBS) and 1X penicillin/streptomycin (P/S) at 37˚C in 5% CO_2_ incubator. Huh7 (Department of Medical Oncology, Beijing You'an Hospital, Capital Medical University) were cultured with DMEM, 10% FBS and P/S at 37˚C in 5% CO_2_ incubator.

The pcDNA3.1 vector containing the human FABP5 cDNA (for overexpression), the empty vector (as an overexpression control), FABP5 short interfering RNA (siRNA; for knockdown), and scramble oligos (as a knockdown control) were synthesized by Hanbio Tech (Shanghai, China). The constructs were transfected into cells using Lipofectamine 3000 (Invitrogen, CA, USA) according to the manufacturer's protocol.

The sequences of siRNA and scramble oligos are listed below

| FABP5-siRNA-F | 5’-GGGAGAGAAGUUUGAAGAATT-3’ |
| --- | --- |
| FABP5-siRNA-R | 5’-UUCUUCAAACUUCUCUCCCTT-3’ |
| FABP5-scramble-F | 5’-UUCUCCGAACGUGUCACGUTT-3’ |
| FABP5-scramble-R | 5’-ACGUGACACGUUCGGAGAATT-3’ |

**Real-time PCR**

Total RNA was extracted from cells using RNAzol Plus Reagent from GenBetter Biotech (Beijing, China). cDNA was prepared using the HiScript III All-in-one RT SuperMix from Vazyme (Nanjing, China). Real-time PCR (RT-PCR) was performed using 2 × Taq Master Mix from Vazyme (Nanjing, China) according to the manufacturer's protocol. The primer sequences for RT-PCR were designed by the authors and chemically synthesized by Tsingke (Beijing, China).

The primer sequences are listed below

| Human-FABP5-F | 5’-CCCTGGGAGAGAAGTTTGAAG-3’ |
| --- | --- |
| Human-FABP5-R | 5’-ATCCCACTCCTGATGCTGAA-3’ |
| Human-β-actin-F | 5’-AGCACTGTGTTGGCGTACAG-3’ |
| Human-β-actin-R | 5’-TGTTTGAGACCTTCAACACCC-3’ |

**CCK8 assay**

Huh7 and HGC-27 cells (both overexpression and knockdown, as well as their respective controls) were suspended with complete mediumand inseeded at a density of 3000 cells per well in a 96-well plate and allowed to grow for 24, 48, 72, and 96 hours. Cell viability was measured using the CCK-8 assay according to the supplier’s protocols (MedChemExpress, NJ, USA).

**Cell migration assay**

We checked the cell migration ability using an in vitro wound healing assay. Cells were suspended with complete medium and inseeded into 6 well culture plates at a density of 1.0 × 10^6^ cells/well. After the cells reached confluence within 24 hours, a single scratch was created in the center of the cell monolayer using a sterile plastic pipette tip. Then, the wells were washed with phosphate-buffered saline (PBS) to remove the detached cells, and the medium was changed to DMEM or RPMI-1640. Images of the wounds were obtained at 0, 24 and 48 hours after making the scratches.

**Transwell migration assay**

Huh7 and HGC-27 cells (both overexpression and knockdown, as well as their respective controls) were suspended in DMEM or RPMI-1640 and seeded at a density of 2.0 × 10^5^ cells in a total volume of 300 μL per insert (8-μm pore size, 24-well insert; Corning, NY, USA). Each insert was pre-coated with 1 mg/mL Matrigel (Corning, NY, USA) before cell seeding. The lower wells were filled with 700 μL of DMEM or RPMI-1640 as a chemoattractant. The Transwell units were incubated at 37˚C in 5% CO_2_ for 24 hours (HGC-27) or 48 hours (Huh7). Migrating cells were fixed with paraformaldehyde for 30 minutes and stained with 0.1% crystal violet for 30 minutes. Cells were counted under high-power magnification (×200).
