# Supplementary material for: Effects of FABP5 Expression on Clinicopathological and Survival Characteristics in Digestive System Malignancies: A Systematic Review and Meta‐Analysis
Source: Cancer Med. 2025 Apr 3;14(7):e70794. doi: 10.1002/cam4.70794 (PMC11966564; doi:10.1002/cam4.70794)
Supplement: Supplementary file 2 — Data S2. [file CAM4-14-e70794-s001.docx]

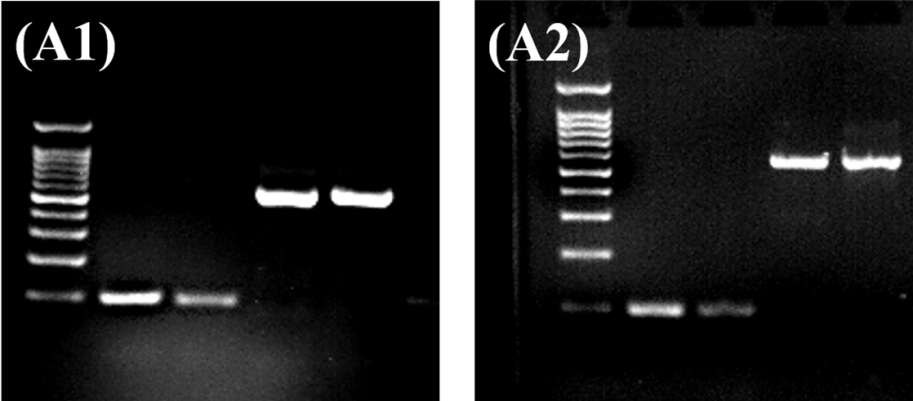


Appendix 5A. Overexpressing cell models validated by Real-time PCR (RT-PCR): (A1) Huh7 cell line; (A2) HGC-27 cell line.


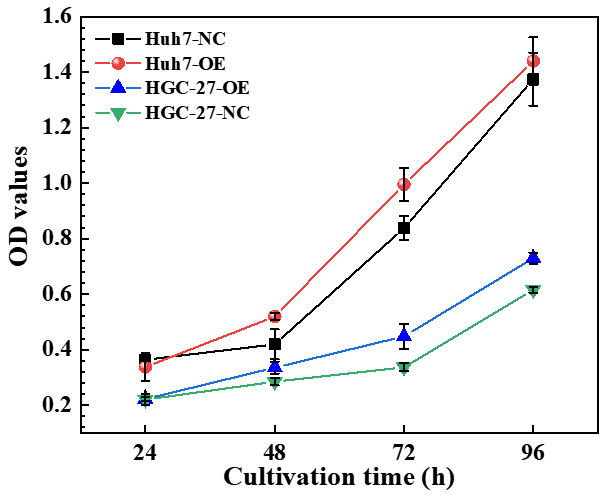


Appendix 5B. Proliferative capacity of Huh7 cell lines (overexpression and control) and HGC-27 cell lines (overexpression and control) validated by CCK8 assay.


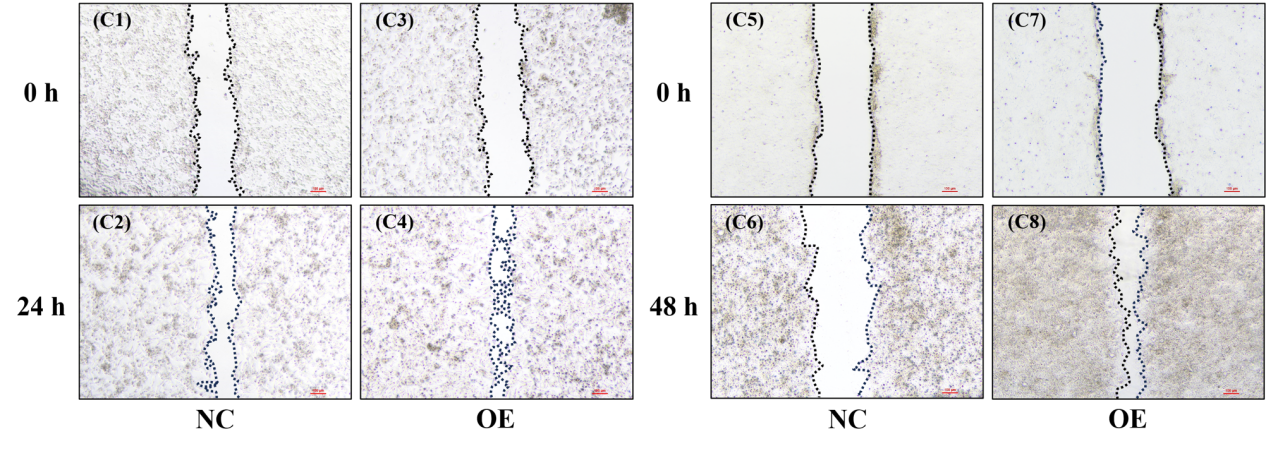


Appendix 5C. Cell migration capability validated by scratch assay:
(C1) Scratch assay of HGC-27 cell line at 0 h (overexpression control);
(C2) Scratch assay of HGC-27 cell line at 24 h (overexpression control);
(C3) Scratch assay of HGC-27 cell line at 0 h (overexpression group);
(C4) Scratch assay of HGC-27 cell line at 24 h (overexpression group);
(C5) Scratch assay of Huh7 cell line at 0 h (overexpression control);
(C6) Scratch assay of Huh7 cell line at 48 h (overexpression control);
(C7) Scratch assay of Huh7 cell line at 0 h (overexpression group);
(C8) Scratch assay of Huh7 cell line at 48 h (overexpression group).


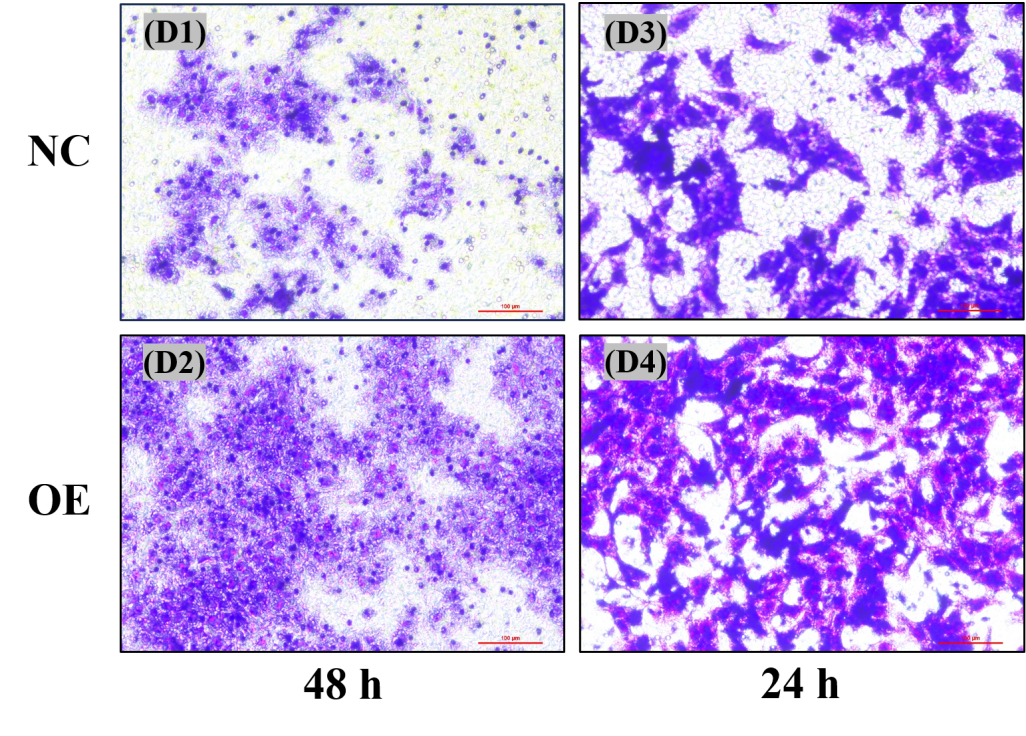


Appendix 6D. Cell invasion capability validated by Transwell assay:
(D1) Huh7 cell line overexpression control group at 48 h;
(D2) Huh7 cell line overexpression group at 48 h;
(D3) HGC-27 cell line overexpression control group at 24 h;
(D4) HGC-27 cell line overexpression group at 24 h.
