# Supplementary material for: Effects of FABP5 Expression on Clinicopathological and Survival Characteristics in Digestive System Malignancies: A Systematic Review and Meta‐Analysis
Source: Cancer Med. 2025 Apr 3;14(7):e70794. doi: 10.1002/cam4.70794 (PMC11966564; doi:10.1002/cam4.70794)
Supplement: Supplementary file 3 — Data S3. [file CAM4-14-e70794-s002.docx]

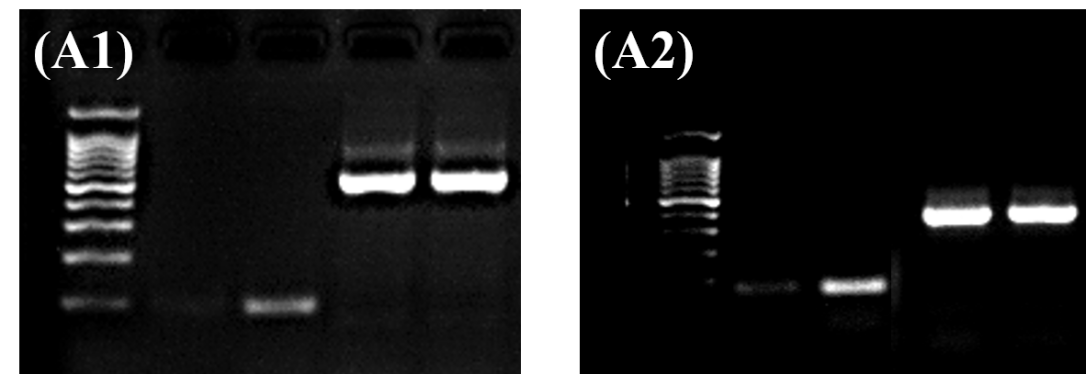


Appendix 6A. Knockdown cell models validated by Real-time PCR (RT-PCR):
(A1) Huh7 cell line; (A2) HGC-27 cell line.


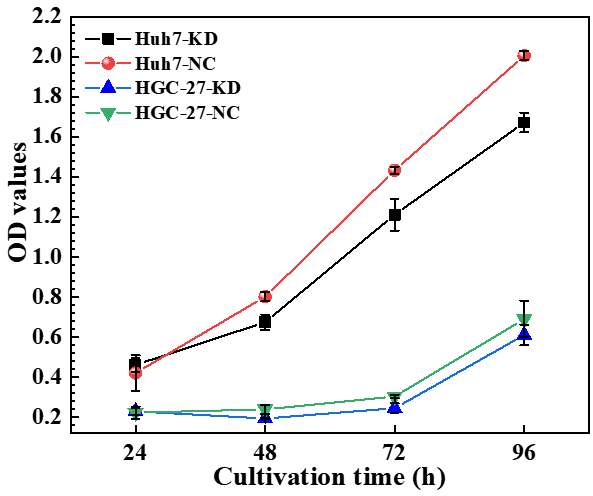


Appendix 6B. Proliferative capacity of Huh7 cell lines (knockdown and control) and HGC-27 cell lines (knockdown and control) validated by CCK8 assay.


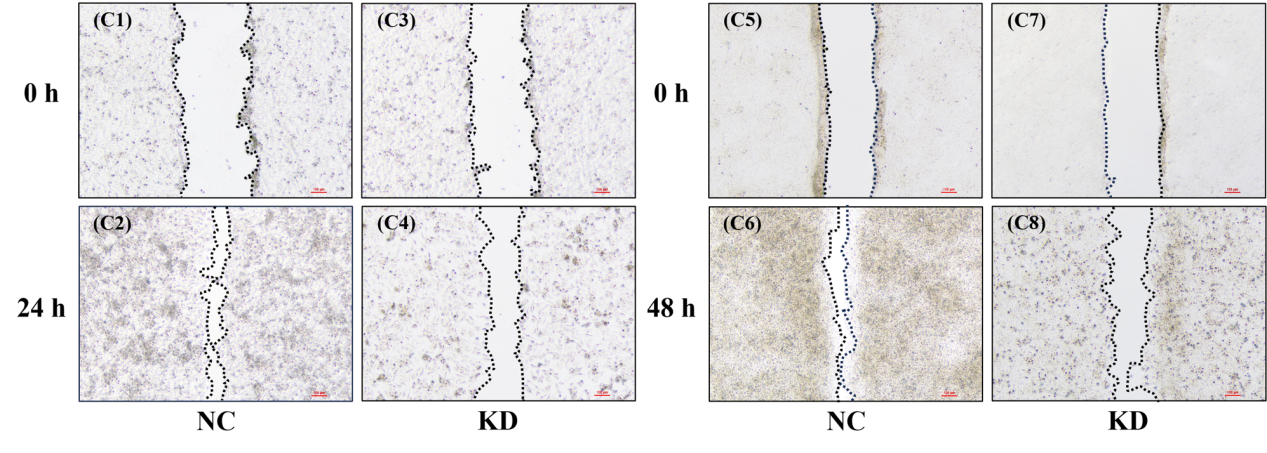
Appendix 6C. Cell migration capability validated by scratch assay:
(C1) HGC-27 cell line (knockdown control) at 0 h;
(C2) HGC-27 cell line (knockdown control) at 24 h;
(C3) HGC-27 cell line (knockdown group) at 0 h;
(C4) HGC-27 cell line (knockdown group) at 24 h;
(C5) Huh7 cell line (knockdown control) at 0 h;
(C6) Huh7 cell line (knockdown control) at 48 h;
(C7) Huh7 cell line (knockdown group) at 0 h;
(C8) Huh7 cell line (knockdown group) at 48 h.


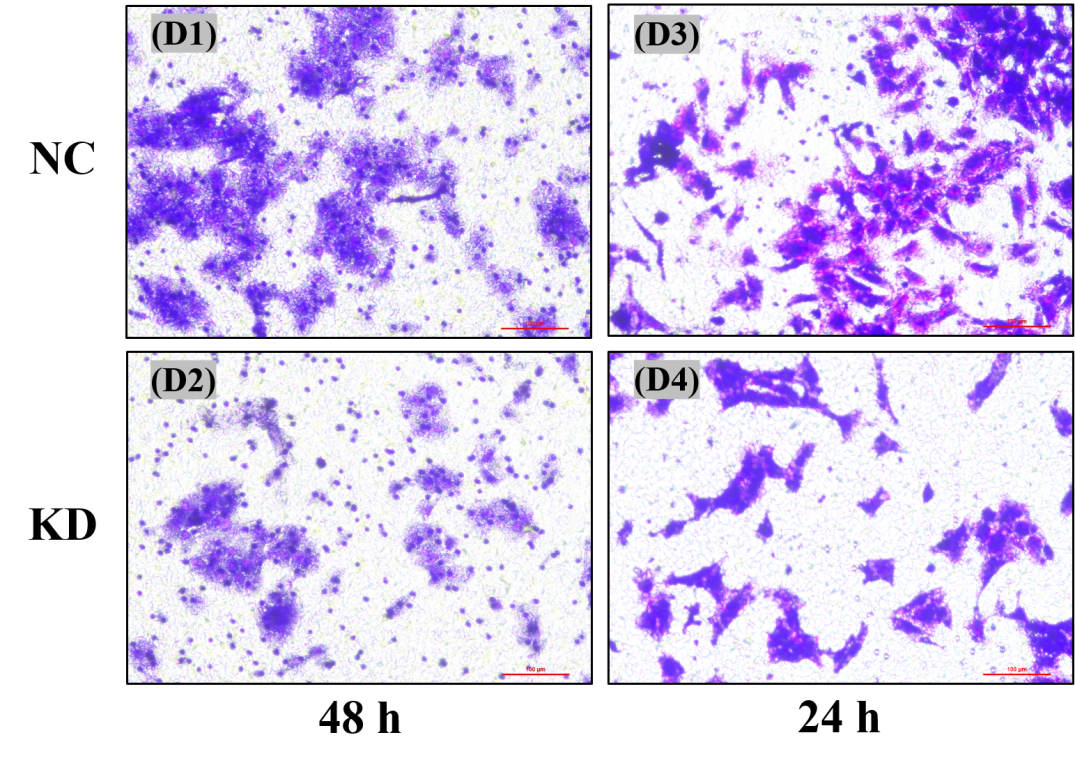


Appendix 6D. Cell invasion capability validated by Transwell assay:
(D1) Huh7 cell line (knockdown control) at 48 h;
(D2) Huh7 cell line (knockdown group) at 48 h;
(D3) HGC-27 cell line (knockdown control) at 24 h;
(D4) HGC-27 cell line (knockdown group) at 24 h.
